# Solutes in water affect the primary cavitation bubble generated by a pulsed erbium-doped yttrium aluminium garnet laser

**DOI:** 10.1007/s10103-024-04257-y

**Published:** 2024-12-18

**Authors:** Maarten Meire, Ben van Aelst, Aldin Sehovic, Shengjile Deari, Matthias Zehnder

**Affiliations:** 1 Ghent University, Ghent, Belgium; 2 University of Zurich, Zurich, Switzerland

**Keywords:** Laser, Root canal, Irrigation, Sodium hypochlorite

## Abstract

Laser-activated irrigation (LAI) of root canal systems depends on the generation of cavitation bubbles in the endodontic irrigant. Physical studies thus far focused on pulse energy, pulse length, frequency, and fiber tip shape, mostly in plain water. This study investigated the effect of endodontically relevant molecules (sodium hypochlorite (NaOCl), 1-hydroxyethylidene-1,1-diphosphonic acid (HEDP), and their combination) in water on physical properties of the resulting solution, and their impact on primary cavitation bubble features. A commercially available 3% NaOCl irrigant was used, as well as an etidronate powder (Dual Rinse HEDP) to be admixed. Physical parameters (density, surface tension, and viscosity) of these solutions were assessed, including HEDP effects in an ascending concentration series of up to 20%. Primary cavitation bubble features (dimensional and temporal) in conjunction with a pulsed erbium-doped yttrium aluminium garnet (Er: YAG) laser equipped with a flat or conical fiber tip were studied in these liquids using a high-speed camera. Solutes increased the solution’s density, surface tension, and viscosity, with an almost linear response to HEDP dosage (Pearson correlation coefficient > 0.95). This reduced the speed of the primary cavitation bubble, and increased its size and lifetime. Increased HEDP concentrations had a pronounced effect on the shape of bubbles generated at the flat tip. NaOCl and HEDP alter the physical properties of water, which, in turn, affect its cavitation behavior.

## Introduction

The treatment concept for teeth with an irreversibly inflamed pulp or a root canal infection is to clean and disinfect the root canal system, followed by root filling or a revascularization procedure [1]. Traditionally, the clinical emphasis was on the mechanical aspects of this treatment. However, the current trend is to reduce tooth substance loss by minimizing mechanical instrumentation, and to rely on the irrigating solutions for cleaning and disinfection [2]. Using chemically active solutions aids in the debridement process [3].

In the search for quicker and more thorough methods of root canal cleansing, laser-assisted irrigation or LAI has garnered considerable interest over the recent years [4]. LAI is based on the absorption of microsecond light pulses in the aqueous root canal irrigant, creating powerful cavitation effects via the generation and subsequent collapse of vapor bubbles [5]. Different concepts and commercially available laser devices exist. They commonly employ a laser with a wavelength that has a high degree of absorption in water [6]. Apart from that, there are different kinds of laser tips, which can either be inserted into the root canals to different lengths, or merely be placed in the access cavity flooded with the irrigant [7]. Moreover, there is a multitude of other factors that are discussed in the context of LAI, such as pulse energy, pulse length, frequency, and wavelength [4].

In vitro assessments of LAI concepts have thus far focused on laser settings, tips, and insertion depths [8, 9]. Frequently water was used as the experimental irrigant to exclude any chemical effects that could influence the result [10]. However, in clinics, root canals are irrigated with sodium hypochlorite (NaOCl) solutions [11]. Even without activation, NaOCl solutions feature unique biolytic properties. They dissolve necrotic tissue and biofilm components that can be contained in root canal systems undergoing/requiring root canal treatment [12, 13]. Interestingly, there appears to by a synergistic effect between the NaOCl in solution and the application of an energy source creating cavitation bubbles [14, 15].

NaOCl is just one solute that can be contained in liquids serving as root canal irrigants. It is known to influence the absorption pattern of an aqueous solution in the ultraviolet region [16]. However, for LAI using erbium lasers, the relevant absorption is taking place in the near-infrared region, therefore NaOCl does not affect the degree of absorption in the irrigant [16]. Apart from absorption patterns, there may be other solution properties that are influenced by the addition of NaOCl (or other solutes) to an aqueous irrigant. Solution density, surface tension, and viscosity all affect the way liquids behave, flow, and undergo phase transformations [17]. Therefore, these solution properties may also influence LAI.

Newer trends in root canal irrigation have aimed to create an all-in-one irrigant by adding a decalcifying agent with short-term NaOCl compatibility [18]. A clinically used and commercially available product is in the form of an etidronate salt, which is added to the NaOCl irrigating solution to form 1-hydroxyethylidene 1,1 diphosphonic acid (HEDP), a mild chelator [18]. This obviously increases solution density, and also increases the surface tension of the combined NaOCl-HEDP solution [19]. It has recently been observed that HEDP also increases the viscosity of NaOCl irrigants [20]. An increased viscosity has been shown to affect the cavitation behavior, albeit in non-Newtonian shear-thickening fluids [21].

It is unclear how the changed solution properties of a NaOCl & HEDP solution affect the cavitation process that is the basis for laser-activated irrigation. We therefore studied primary cavitation generated by a pulsed Er: YAG laser in clinically recommended endodontic irrigants with/without HEDP, and then followed the observed phenomena further by increasing HEDP content beyond clinically recommended levels.

## Materials and methods

### Solutions

Deionized water (Milli-Q, Merck, Rahway, NJ) was used as a control in all the experiments. The endodontic NaOCl solution under investigation was CanalPro 3% (Coltène, Altstätten, Switzerland, LOT 20222889 and 20222937). The etidronate powder that is advocated as a decalcifying add-on to NaOCl solutions was Dual Rinse HEDP (Medcem, Weinfelden, Switzerland, LOT DR230628) The combined NaOCl & HEDP solutions were mixed freshly before the experiments, by adding the adequate amount of powder (wt/wt) to the liquid necessary for the individual assessment. Powder to liquid mixtures were prepared using a precision balance (PM300, Mettler-Toledo, Greifensee, Switzerland). All the experiments described below were performed at an ambient temperature of 22–23 °C. The NaOCl solution that had be stored in the refrigerator was warmed in a water bath to 20 °C before use.

To assess whether there was a specific influence of the etidronate powder (Dual Rinse HEDP, Medcem) dissolved in the NaOCl solution, a similar mixture (9.1 g of NaOCl solution & 0.9 g of powder) was prepared using table salt (NaCl). Moreover, to then assess the impact of dissolved etidronate (HEDP) beyond clinical dosages on physico-chemical solution properties and cavitation bubble features, a series of solution was prepared containing (wt/wt) 0%, 5%, 10% and 20% Dual Rinse HEDP (Medcem) in the NaOCl solution (Canal Pro 3%, Coltène).

### Chemical and physical assessments

The content of available chlorine in the solutions was measured by iodometric titration. The solution was spiked with potassium iodide (PanReac AppliChem, Darmstadt, Germany), and rendered acidic by adding HCl (Emsure 1.00316.1011, Merck). The liberated iodine was then titrated immediately using a 0.1 M sodium thiosulfate solution (PanReac AppliChem 186987.1211) in a titration apparatus (665 Dosimat, Metrohm, Herisau, Switzerland). Hydrogen ion activity was assessed using a calibrated electrode (6.0228.010, Metrohm) attached to a pH measuring device (727 pH lab, Metrohm).

Surface tension was measured in a pendant drop shape analyzer (DSA100, KRÜSS GmbH, Hamburg, Germany) at an ambient temperature of 22 °C. Surface tension was calculated based on the drop shape in relation to needle diameter (0.98 mm) and density of the liquid by the proprietary software. Liquid density was assessed using a high-precision balance (AT261, Mettler Toledo) by measuring the weight of 1.0 mL of the solution.

Viscosity of the solutions was determined in a rotational viscometer for low viscosity assessments (ViscoQC 300, Anton Paar, Buchs, Switzerland) equipped with a proprietary measuring bob (B-DG26) in a corresponding measuring cup (C-DG26). Viscosity measurements were performed at a rotational speed of 100 rpm in a controlled temperature environment of 20 °C (Checktemp 1, Hanna Instruments, Smithfield, RI) in a water bath (M3, Lauda, Königshofen, Germany).

### Analysis of primary cavitation at the laser tip

A 2940 nm erbium-doped yttrium aluminium garnet (Er: YAG) laser (Skypulse, Fotona, Lljubliana, Slovenia) was equipped with a H14 handpiece holding either a flat or a conical fiber tip (SWEEPS 400/14, Fotona). The tip was placed in an optical glass cuvette (28 × 28 × 35 mm, Hellma, Müllheim, Germany) containing the test or control solutions. It was placed 9.5 mm below the liquid surface. For calibration purposes, a silicone stopper was attached to the fiber tip. Its diameter was measured with a digital caliper. The laser was used in SSP mode (pulse length of 50 µs) at a frequency of 15 Hz, and pulse energy of 40 mJ. A high-speed camera system (Photron SA-X, Tokyo, Japan) was mounted in front of the cuvette and high-speed recordings of 5 s were made. A lens system allowed zooming in on the fiber tip and surrounding liquid. The high-speed camera settings were as follows: frame rate: 81,000 frames per s, exposure time: 1/93,237 s, image resolution: 512 × 256 pixels. The proprietary FASTCAM viewer software PFV4 (Photron) was used to record and analyze the primary cavitation. The following primary cavitation bubble features were determined:

- time to maximal dimension: time (in ms) from start formation to its maximum dimension;
- lifetime: time (in ms) from formation to implosion of the primary bubble;
- distance between the laser tip and the distal end of the bubble: the portion of the bubble (in mm) in front of the laser tip.
- the maximum length (in mm).
- the maximum width (in mm).
- the total area (in mm²).

One parameter, bubble expansion speed, denoting the speed of expansion (in mm/ms) of the primary bubble, was calculated as follows: distance between the laser tip and the distal end of the bubble divided by time to maximal dimension.

Distance measurements were all performed at maximum bubble dimension, using the measuring tool of the PFV software. For surface measurements, the frame with the maximum bubble dimension was imported in Fiji and analyzed using the polygon selection tool and area measurement function. In both instances, the silicone stopper was used for image calibration. Duration was measured based on the time (in ms) as indicated per frame in the FastCam Viewer. All measurements were carried out on 3 consecutive primary cavitation bubbles per condition.

### Data presentation and analysis

All measurements were done in triplicate (*n* = 3). The data presented here all represent means and standard deviations, indicating the measurement error. The strength of the linear correlation between HEDP concentration in the endodontic NaOCl irrigant and physical parameters of the resulting solutions as well as primary cavitation bubble features was explored by linear regression analysis using a software program (JMP Pro 17, SAS Institute, Cary, NC), and the Pearson correlation coefficients (PCCs) are reported.

## Results

### Observations with clinically used solutions

#### Chemical and physical properties

The commercially available endodontic NaOCl solution in this study had an active chlorine content (3.7% Cl_2_, equaling 3.8% NaOCl) that was higher than the 3% indicated on its label (Table 1). All the solutes in water, i.e. NaOCl, NaCl, and HEDP increased its density, surface tension, and viscosity (Table 1). The addition of HEDP to the NaOCl irrigant increased viscosity considerably more than did the table salt (NaCl) (Table 1).

**Table 1:** Characterization of the solutions used in this study (triplicate measurements, means ± standard deviations, pH ranges)

Variable	Deionized water (Milli-Q)	CanalPro 3%	CanalPro 3% & 9% NaCl*	CanalPro 3% & 9% HEDP*
NaOCl (wt%)	0.00 ± 0.00	3.89 ± 0.00	3.56 ± 0.02	3.41 ± 0.08
pH range	N/A	12.1–12.2	12.1–12.9	11.4–11.6
Density (g/mL)	1.00 ± 0.00	1.06 ± 0.00	1.12 ± 0.00	1.13 ± 0.00
Surface tension (mN/m)	72.9 ± 0.2	75.2 ± 0.2	78.4 ± 0.2	79.3 ± 0.1
Viscosity (cP)	0.99 ± 0.01	1.25 ± 0.05	1.48 ± 0.02	1.77 ± 0.01

* 0.9 g of the respective salt was mixed into 9.1 g of the NaOCl solution. N/A: not applicable

#### Cavitation analysis

Table 2 summarizes the primary cavitation bubble features generated by the Er: YAG laser when clinically used endodontic irrigants were compared to deionized water.

**Table 2:** Primary cavitation bubble characteristics observed with clinically used endodontic irrigants as compared to deionized water (triplicate measurements, means ± standard deviations)

Laser tip	Bubble parameter	Deionized water (Milli-Q)	CanalPro 3%	CanalPro 3% & 9% NaCl	CanalPro 3% & 9% HEDP
Flat	Time to max. dimensions (ms)	0.14 ± 0.01	0.14 ± 0.01	0.15 ± 0.00	0.15 ± 0.01
	Time to implosion (ms)	0.26 ± 0.01	0.25 ± 0.01	0.26 ± 0.00	0.27 ± 0.00
	Distance from laser tip to tip of bubble (mm)	3.21 ± 0.01	3.12 ± 0.01	3.10 ± 0.05	3.02 ± 0.01
	Maximum bubble length (mm)	3.57 ± 0.03	3.63 ± 0.14	3.95 ± 0.03	3.88 ± 0.05
	Maximum bubble width (mm)	1.85 ± 0.02	1.76 ± 0.04	1.84 ± 0.02	1.87 ± 0.07
	Bubble area (mm²)	5.16 ± 0.10	5.18 ± 0.15	5.90 ± 0.09*	6.08 ± 0.15
	bubble expansion speed (mm/ms)	22.30 ± 1.06	22.33 ± 1.17	20.94 ± 0.32	19.84 ± 0.87
Conical	Time to max. dimensions (ms)	0.16 ± 0.01	0.18 ± 0.01	0.17 ± 0.01	0.18 ± 0.01
	Time to implosion (ms)	0.26 ± 0,01	0.28 ± 0.01	0.28 ± 0.03	0.30 ± 0.01
	Distance from laser tip to tip of bubble (mm)	1.41 ± 0.04	1.41 ± 0.04	1.38 ± 0.08	1.45 ± 0.04
	Maximum bubble length (mm)	2.63 ± 0.06	2.61 ± 0.15	2.58 ± 0.15	2.67 ± 0.05
	Maximum bubble width (mm)	2.02 ± 0.06	2.19 ± 0.18	2.16 ± 0.27	2.33 ± 0.05
	Bubble area (mm²)	4.25 ± 0.23	4.66 ± 0.61	4.52 ± 0.80	5.03 ± 0.15
	Bubble expansion speed (mm/ms)	9.03 ± 0.42	7.94 ± 0.09	8.21 ± 0.24	8.20 ± 0.48

**Fig. 1:**
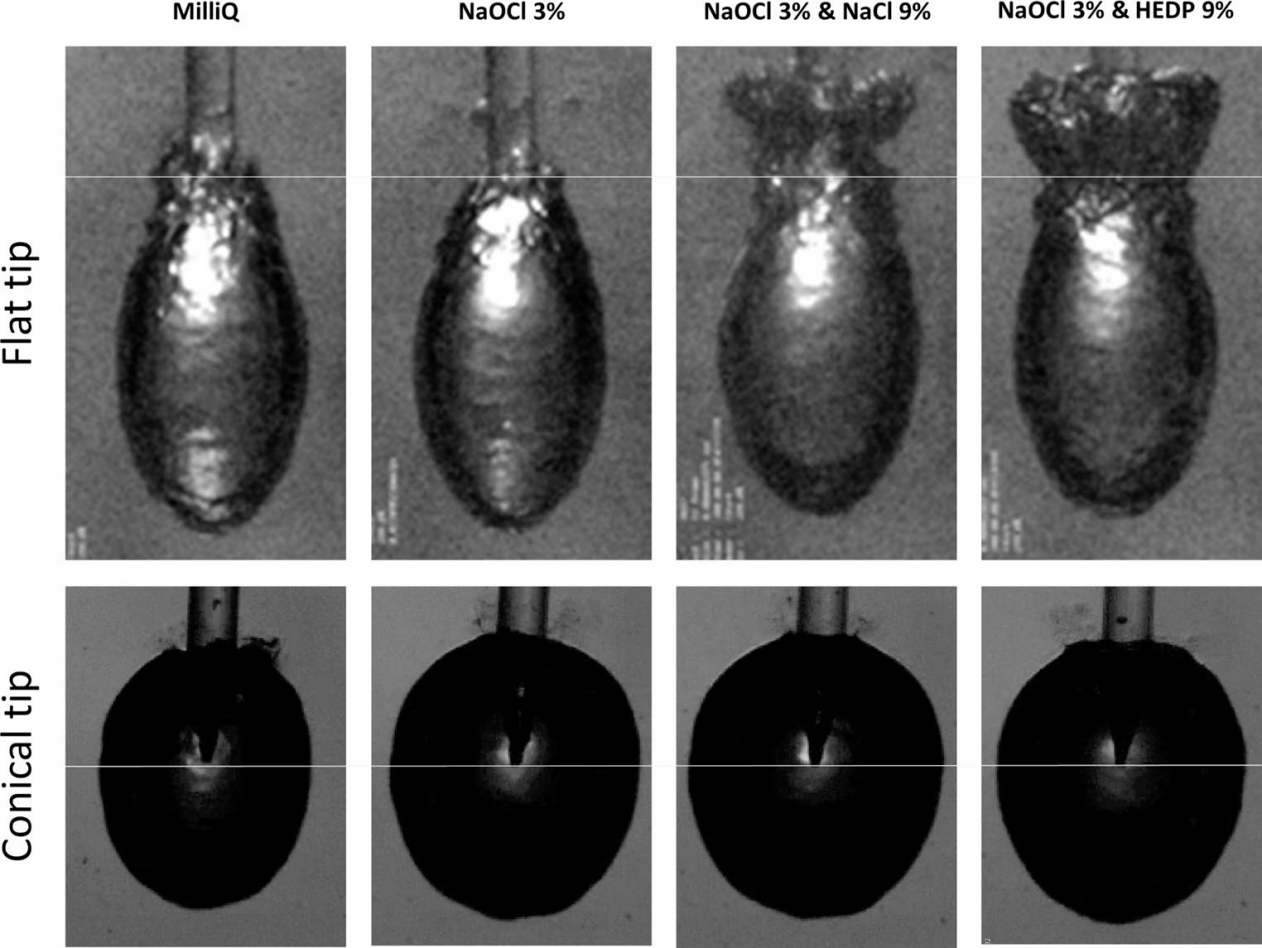
High-speed images of primary cavitation bubbles (captured at maximum volume) in clinically used endodontic irrigants. Upper panels represent cavitation at a flat tip, lower panels at a conical tip. Yellow lines indicate the position of the fiber tip end

With the flat tip, there was little effect of the solutes on the time to maximum bubble dimension and the bubble lifetime. Bubble dimensions however gradually increased: from 5.16 ± 0.10 mm² to 5.18 ± 0.15 mm² with NaOCl, to 5.90 ± 0.09 mm² with NaOCl & NaCl, and finally to 6.08 ± 0.15 mm² with NaOCl & HEDP in the solution (Table 2). Bubble expansion speed decreased slightly in the solutions combining NaOCl with NaCl or HEDP. When NaCl or HEDP were added to the NaOCl irrigant, bubble extension beyond the fiber tip decreased, and the overall bubble shape changed from an ellipsoid to a waisted shape with a distinct tail end (Fig. 1).

With the conical laser tip, addition of solutes led to a slight increase in time to maximum bubble dimension and bubble lifetime. Bubble dimensions also increased gradually: from 4.25 ± 0.23 mm² to 4.66 ± 0.61 mm² with NaOCl, to 4.52 ± 0.80 mm² with NaOCl & NaCl, and finally to 5.03 ± 0.15 mm² with NaOCl & HEDP in the solution (Table 2). The bubble shape however seemed to not be altered significantly by the solutes (Fig. 1). Bubble expansion speed decreased slightly in the solutions combining NaOCl and NaCl or HEDP.

### Observations with increasing HEDP content

#### Chemical and physical properties

Because the solutes in water under investigation had complex and non-uniform effects on the physical properties of the solutions under investigation, further experiments were performed with an ascending concentration of HEDP in the endodontic NaOCl solution. Within the concentration range under investigation, the addition of HEDP had a straight dose-dependent positive effect on solution density, surface tension and viscosity (PCC > 0.95, Table 3).

**Table 3:** Physical characterization of the endodontic NaOCl solution used in this study spiked with increasing amounts of HEDP (triplicate measurements, means ± standard deviations) including the linear correlation between HEDP concentration and these features

Concentration of HEDP in the CanalPro 3% solution	Density (g/mL)	Surface tension (mN/m)	Viscosity (cP)
0%	1.06 ± 0.00	75.2 ± 0.2	1.25 ± 0.05
5%	1.07 ± 0.00	77.6 ± 0.2	1.45 ± 0.04
10%	1.13 ± 0.00	79.5 ± 0.1	1.92 ± 0.06
20%	1.22 ± 0.01	81.3 ± 0.3	3.44 ± 0.07
PCC*	0.98	0.97	0.97

*Pearson correlation coefficient

#### Cavitation analysis

**Fig. 2:**
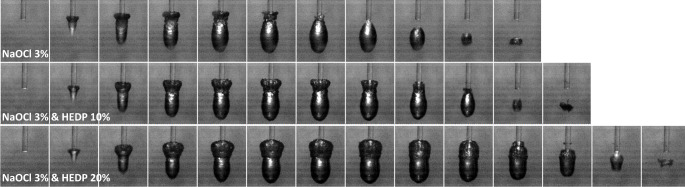
High-speed image sequences of primary cavitation in NaOCl 3% with increasing HEDP content

**Fig. 3:**
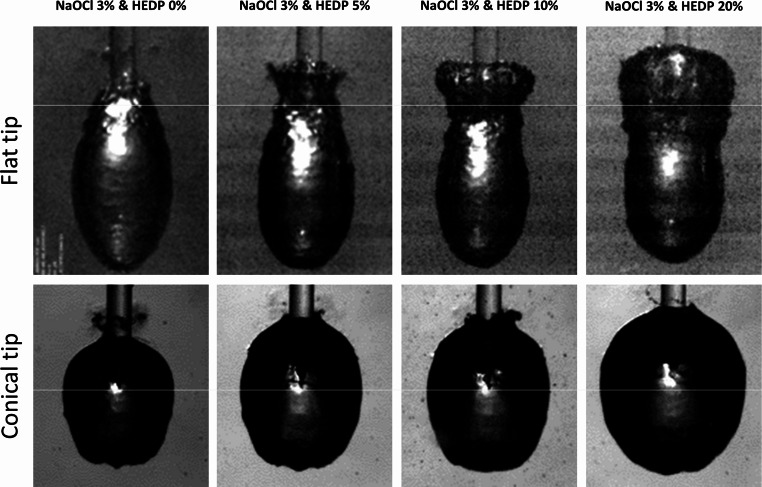
High-speed images of primary cavitation bubbles (captured at maximum volume) in NaOCl 3% with increasing HEDP content. Upper panels represent cavitation at a flat tip, lower panels at a conical tip. Yellow lines indicate the position of the fiber tip end

An increasing HEDP concentration in the NaOCl irrigant under investigation had a dose-dependent effect on primary cavitation bubbles, which was similar between the flat and the conical laser tip (Table 4). There was an increase in time to maximal dimensions, time to implosion, maximum bubble length, width, and area. The distance between laser tip and distal bubble end increased in the case of a conical, and decreased in case of a flat tip. A decrease in bubble expansion speed was apparent with both tips (PCC < -0.84). With the flat laser tip, the change in bubble shape, with the formation of a pronounced tail part at the distal aspect of the ellipsoid, was again apparent. With increasing HEDP concentration, the proportion of this tail part increased at the expense of the main ellipsoid (Fig. 2). With the conical tip, the bubble shape was not significantly affected by the solute concentration (Fig. 3).

**Table 4:** Exploration of the effect of increasing HEDP concentration in the endodontic NaOCl solution (CanalPro) on the primary cavitation bubble characteristics according to laser tip design (triplicate measurements, means ± standard deviations)

Laser tip	Bubble parameter	CanalPro 3%	CanalPro 3% & 5% HEDP	CanalPro 3% & 10% HEDP	CanalPro 3% & 20% HEDP	PCC*
Flat	Time to max. dimensions (ms)	0.14 ± 0.01	0.15 ± 0.01	0.15 ± 0.00	0.17 ± 0.00	0.88
	Time to implosion (ms)	0.25 ± 0.01	0.26 ± 0.00	0.26 ± 0.00	0.32 ± 0.01	0.93
	Distance from laser tip to tip of bubble (mm)	3.12 ± 0.01	3.09 ± 0.03	2.95 ± 0.01	2.95 ± 0.05	− 0.83
	Maximum bubble length (mm)	3.63 ± 0.14	3.89 ± 0.01	3.83 ± 0.02	4.07 ± 0.08	0.83
	Maximum bubble width (mm)	1.76 ± 0.04	1.83 ± 0.03	1.82 ± 0.07	2.14 ± 0.06	0.89
	Bubble area (mm²)	5.18 ± 0.15	5.58 ± 0.09	5.86 ± 0.17	7.18 ± 0.32	0.96
	Bubble expansion speed (mm/ms)	22.33 ± 1.17	20.35 ± 1.12	19.91 ± 0.07	17.09 ± 0,26	− 0.93
Conical	Time to max. dimensions (ms)	0.14 ± 0.00	0.15 ± 0.01	0.16 ± 0.01	0.19 ± 0.01	0.88
	Time to implosion (ms)	0.25 ± 0.01	0.28 ± 0.03	0.29 ± 0.01	0.35 ± 0.03	0.91
	Distance from laser tip to tip of bubble (mm)	1.43 ± 0.03	1.49 ± 0.04	1.5 ± 0.03	1.63 ± 0.09	0.85
	Maximum bubble length (mm)	2.56 ± 0.10	2.77 ± 0.12	2.75 ± 0.05	3.05 ± 0.24	0.80
	Maximum bubble width (mm)	2.01 ± 0.07	2.18 ± 0.17	2.24 ± 0.05	2.65 ± 0.30	0.84
	Bubble area (mm²)	4.19 ± 0.12	4.88 ± 0.46	5.14 ± 0,26	6.43 ± 1.23	0.82
	Bubble expansion speed (mm/ms)	10.55 ± 0.19	9.81 ± 0.77	9.59 ± 0.49	8.62 ± 0.31	− 0.85

*Pearson correlation coefficient

## Discussion

This is the first study to explore the influence of solutes on Er: YAG laser-induced cavitation via their effects on physical properties of the aqueous irrigants they are contained in. The data prove that the studied solutes do affect the cavitation process in LAI via the alteration of physical solution properties, and suggest that investigations of cavitation phenomena and their simulated clinical effects should take place in the appropriate solution.

While the current study revealed some new and interesting observations related to endodontic irrigants and their cavitation behavior, it has clear limitations. First and foremost, it remains unknown how the current findings relate to clinics, as they were obtained in a laboratory environment. The question is whether the observed differences in primary cavitation might also impact the cleaning action inside the root canal system, the risk of irrigant extrusion, or other clinical features. Furthermore, the focus of this study was on the primary cavitation bubble. The cleaning action inside the canal system is not a direct effect of the primary cavitation bubble itself, but rather the result of fluid streaming induced by the primary bubble’s oscillations [22]. The effect of solutes on these secondary cavitation phenomena, such as the appearance of smaller bubbles throughout the entire canal during primary bubble implosion [23], was not studied here.

Despite this study’s limitations, it appears sound to draw a few conclusions and discuss these observations. First, the NaOCl content of the endodontic solution under investigation was above its labeled strength. This is contrary to other random samples of other NaOCl solutions, which usually contained less available chlorine than advocated [20, 24]. While the exact chlorine content of an endodontic irrigant may not be as clinically relevant as some clinicians believe [25], it should nevertheless be criticized that such essential endodontic products are not labeled more accurately.

Surface tension and viscosity are related features of a liquid. Both are driven by forces between the molecules within the fluid [17]. However, surface tension is a static feature, while viscosity is more complex and dynamic. Interestingly, NaOCl solutions containing surfactants may have a reduced surface tension compared to plain counterparts, while their viscosity remains unaffected [20]. This study followed the idea that a change in physical fluid parameters could also affect LAI-induced cavitation in the Newtonian liquids under investigation [21]. Although it is known that increased surface tension and fluid viscosity (as induced by the addition of solutes) counteract vapor bubble formation [26], and result in smaller or more spherical bubbles, the opposite was observed in the present study. Cavitation is a complicated physical process, in which other parameters and liquid properties come into play. These include liquid temperature, boiling point, vapor pressure, etc [26]. Although liquid temperature was not measured during the experiments, the volume of liquid in the cuvette was large compared to the energy delivered, and the laser was activated only a few seconds for each test condition. Therefore, we think that liquid temperature had limited influence on the cavitation process during the experiments.

The majority of studies that have focused on the cavitation generated by erbium lasers in the context of root canal cleaning used water or distilled water as the test solution [6, 22, 27–31]. The present data suggest that the use of water in these studies may not accurately reflect the clinical conditions, where laser-activation is typically executed in endodontic irrigants such as NaOCl, EDTA, or a NaOCl & HEDP combination. The addition of NaOCl and HEDP to water increased density of the resulting liquid, as well as its surface tension and viscosity. This is in line with published data [20]. These physical alterations affected the primary cavitation induced by an Er: YAG laser. Bubbles in a 3% NaOCl & 9% HEDP solution had a longer lifetime and a greater volume compared to bubbles in deionized water, or in plain 3% NaOCl. In case of a flat fiber tip, bubble shape was also different. With HEDP in the irrigating solution, the effects on bubble life time, bubble volume and bubble shape became even more pronounced with increasing concentration. Although differences in primary cavitation between distilled water and 3% NaOCl were minor around flat fiber tips (Fig. 1), differences around conical tips were more pronounced, with cavitation bubbles being bigger and existing longer. With the clinically used endodontic irrigants, the greatest differences were observed between distilled water and a solution with 3% NaOCl & 9% HEDP. In case of both flat and conical fiber tips, the primary cavitation bubble was larger in this solution (Table 2). In the case of the flat tip, the bubble shape was also affected, with a smaller part of the bubble extending in front of the fiber tip, and a tail-like structure developing at the proximal end of the bubble. This effect became even more visible in the dose-response tests with increasing HEDP concentrations beyond the clinically recommended dosage, where the backward projection of the bubble becoming more important, and a shape being waisted rather than elliptical (Fig. 2). Whether these phenomena affect the clinical effectiveness of a combined NaOCl & HEDP irrigant remains to be investigated. The effects of endodontic irrigation are only partly physical. Chemical aspects exerted by solutes in endodontic irrigants, such as the proteolytic properties of NaOCl, play a key role in this context, as do the decalcifying features of chelators such as EDTA or HEDP [3].

It is well-established that the extension of the bubble’s lifetime influences the energy density of the cavitation process. Gregorcic and co-workers demonstrated generation of shock waves upon bubble collapse in an infinite liquid, but not in a tooth model [22]. This was due to the longer oscillation time of the primary bubble. As the process of bubble formation and collapse due to addition of solutes is prolonged, as in the present study, the mechanical energy associated with the bubble kinetics is spread over a longer time frame, making the process potentially less violent. Theoretically, this could affect the cleaning action negatively. On the other hand, it was also noted that addition of solutes resulted in greater bubble volume, which may increase the emitted energy when the bubble collapses [32], or result in more pronounced fluid streaming within the canal, thereby enhancing biofilm removal.

Future studies should study the phenomena described here in a clinically simulated environment. LAI cleaning of organic and inorganic remnants in a real or simulated root canal system should be compared between water, NaOCl, and a combined NaOCl & HEDP solution to weigh physical against chemical effects.

In conclusion, this study showed that solutes in water influence Er: YAG laser-induced primary cavitation in a dose-dependent manner, resulting in prolongation of the lifetime and increase in size of the primary bubble, and a change in the bubble shape at the tip of a flat fiber.

## Data Availability

No datasets were generated or analysed during the current study.
